# Systemic and Extraradicular Bacterial Translocation in Apical Periodontitis

**DOI:** 10.3389/fcimb.2021.649925

**Published:** 2021-03-19

**Authors:** María José Bordagaray, Alejandra Fernández, Mauricio Garrido, Jessica Astorga, Anilei Hoare, Marcela Hernández

**Affiliations:** ^1^ Laboratory of Periodontal Biology, Faculty of Dentistry, Universidad de Chile, Santiago, Chile; ^2^ Department of Conservative Dentistry, Faculty of Dentistry, Universidad de Chile, Santiago, Chile; ^3^ Faculty of Dentistry, Universidad Andres Bello, Santiago, Chile; ^4^ Department of Pathology and Oral Medicine, Faculty of Dentistry, Universidad de Chile, Santiago, Chile; ^5^ Laboratory of Oral Microbiology, Faculty of Dentistry, Universidad de Chile, Santiago, Chile

**Keywords:** periapical periodontitis, periapical lesion, *Porphyromonas*, bacterial translocation, peripheral blood mononuclear cells (PBMC)

## Abstract

Apical periodontitis is an inflammatory disease of microbial etiology. It has been suggested that endodontic bacterial DNA might translocate to distant organs *via* blood vessels, but no studies have been conducted. We aimed first to explore overall extraradicular infection, as well as specifically by *Porphyromonas* spp; and their potential to translocate from infected root canals to blood through peripheral blood mononuclear cells. In this cross-sectional study, healthy individuals with and without a diagnosis of apical periodontitis with an associated apical lesion of endodontic origin (both, symptomatic and asymptomatic) were included. Apical lesions (N=64) were collected from volunteers with an indication of tooth extraction. Intracanal samples (N=39) and respective peripheral blood mononuclear cells from apical periodontitis (n=14) individuals with an indication of endodontic treatment, as well as from healthy individuals (n=14) were collected. The detection frequencies and loads (DNA copies/mg or DNA copies/μL) of total bacteria, *Porphyromonas endodontalis* and *Porphyromonas gingivalis* were measured by qPCR. In apical lesions, the detection frequencies (%) and median bacterial loads (DNA copies/mg) respectively were 70.8% and 4521.6 for total bacteria; 21.5% and 1789.7 for *Porphyromonas endodontalis;* and 18.4% and 1493.9 for *Porphyromonas gingivalis*. In intracanal exudates, the detection frequencies and median bacterial loads respectively were 100% and 21089.2 (DNA copies/μL) for total bacteria, 41% and 8263.9 for *Porphyromonas endodontalis*; and 20.5%, median 12538.9 for *Porphyromonas gingivalis.* Finally, bacteria were detected in all samples of peripheral blood mononuclear cells including apical periodontitis and healthy groups, though total bacterial loads (median DNA copies/μL) were significantly higher in apical periodontitis (953.6) compared to controls (300.7), p<0.05. *Porphyromonas endodontalis* was equally detected in both groups (50%), but its bacterial load tended to be higher in apical periodontitis (262.3) than controls (158.8), p>0.05; *Porphyromonas gingivalis* was not detected. Bacteria and specifically *Porphyromonas* spp. were frequently detected in endodontic canals and apical lesions. Also, total bacteria and *Porphyromonas endodontalis* DNA were detected in peripheral blood mononuclear cells, supporting their plausible role in bacterial systemic translocation.

## Introduction

Apical periodontitis (AP) is the inflammatory destruction of the apical periodontium as the result of endodontic infection. The hallmark of AP is the development of an osteolytic apical lesion of endodontic origin (ALEO). From an epidemiological point of view, AP represents a highly frequent cause of tooth loss and a non-classic risk factor for several non-communicable diseases (NCD), in part, by inducing low-grade systemic inflammation (Garrido et al., 2019). AP can vary over time between two clinical entities, symptomatic and asymptomatic apical periodontitis (SAP and AAP, respectively), depending on the dynamic balance between bacterial consortia and the host’s response (Hsiao et al., 2012). Among them, the former is considered an immunologically exacerbated stage (Veloso et al., 2020).

ALEOs result from the direct communication between the former sterile pulp tissue and the oral microbiota, often caused by dental caries. The endodontic pathogens organize in multispecies biofilm communities within the root canal, favoring the selection of Gram-negative anaerobic bacteria (Sakko et al., 2016). Though a substantial heterogeneity can be found among geographically diverse populations the black-pigmented anaerobes, *Porphyromonas endodontalis* and *Porphyromonas gingivalis*, are key pathogens in light of their high prevalence within the root canals, prominent virulence factors and/or significance in the bacterial community’s stability and virulence (Rôças and Siqueira, 2018).

Bacterial translocation involves the circulation of bacteria and/or their immunogenic products, such as DNA, even without overt infection or clinical signs (Kane et al., 1998; Martinez-Martinez et al., 2009; Ibrahiem et al., 2011). ALEOs might provide a direct pathogenic pathway toward general circulation provoking low-grade systemic inflammation and associated NCD risk. In fact, extraradicular infections have been increasingly identified during the last years, suggesting an initial step in bacterial dissemination beyond the tooth structure, in which they can reach the blood vessels from the apical granuloma (Sunde et al., 2000; Buonavoglia et al., 2013). Furthermore, evidence of endodontic bacterial translocation is indirectly supported by the well-documented enrichment of oral bacterial DNA in diseased peripheral tissues (i.e. atheroma plaques, arthritic joints) (Reichert et al., 2013; Chhibber-Goel et al., 2016; Berthelot and Wendling, 2020). It has been proposed that periodontal bacteria can be transported to distant tissues internalized in immune cells in arthritis (Martinez-Martinez et al., 2009; Ibrahiem et al., 2011), involving mononuclear cell activation, production of pro-inflammatory cytokines, and risk for future NCD complications (Sahingur et al., 2010; Rodriguez-Laiz et al., 2019). Up to now, reports of extraradicular endodontic infection are limited, whereas further bacterial outreach to blood remains unknown. We aimed first to explore overall extraradicular infection, as well as specifically by *Porphyromonas* spp; and their potential to translocate from infected root canals to the blood through peripheral blood mononuclear cells (PBMCs).

## Material and Methods

### Study Design

Cross-sectional. The study was approved by the Ethics-Scientific Committee of the Central Metropolitan Health Service (N 2017/70) and from the Faculty of Dentistry, Universidad de Chile (N 2016/08). The objectives and procedures of the study were informed to the participants and all of them signed the informed consent or its corresponding forms in the case of underage participants. All procedures followed the ethical standards of the institutional and/or national research committee and with the 1964 Helsinki declaration and its later amendments or comparable ethical standards.

### Patient Recruitment

Patients aged between 15 and 40 years old consulting at the dental clinic, School of Dentistry, Universidad de Chile and the Clinic of Surgery, Faculty of Dentistry, Universidad Andrés Bello, Santiago, Chile, were enrolled between 2016 and 2019 if they were otherwise healthy and had a clinical diagnosis of AP in the presence of a radiographic apical radiolucency due to extensive caries in a tooth with negative sensitivity pulpal test with no previous endodontic intervention. SAP and AAP were diagnosed when clinical symptoms in response to percussion were present or absent, respectively, according to previously defined diagnostic terminology (Gutmann et al., 2009). Controls met the same criteria and the absence of any tooth with ALEOs. Exclusion criteria were obesity (body mass index ≥30 kg/m^2^), pregnancy, moderate to severe periodontitis, and antibiotic and/or anti-inflammatory drug consumption 3 months before the study (Garrido et al., 2019).

### Sample Collection

Periapical tissue samples were collected from individuals having indication of tooth extraction. Tissue samples from ALEOs (n=64), including AAP (n=29) and SAP (n=35), or healthy periodontal ligaments (HPL) as negative controls (n=9), were obtained by surgical separation of the tissue from the root surface with sterile curettes. Then the samples were washed with 3 mL of sterile NaCl solution before their storage in 100 μL of RNAlater (Qiagen, Valencia, CA, USA) at -80°C until processed for tissue homogenization.

Root canal exudates and the respective blood samples were collected from AP patients having indication of endodontic treatment. For intracanal exudate sampling (N=39; AAP=29 and SAP=10), the involved tooth was isolated with a rubber dam and disinfected with 70% ethanol solution. After coronal access, sterile paper points (Maillefer^®^) were inserted into the infected root canal for 1 minute, transported in a vial containing 1 mL of cold sterilized reduced transport fluid (RTF), and stored at -80°C for posterior analysis. Blood samples were collected from a subset of AP patients (n=14) and also from healthy control volunteers (n=14) by venipuncture of the antecubital vein (Fouad et al., 2002; Rocas and Siqueira, 2011). PBMCs were isolated using Ficoll-Paque Premium 1.073 (GE Healthcare^®^), following the manufacturer’s instructions.

### DNA Extraction

Apical tissue samples were homogenized in a lysis buffer with lysozyme 20 mg/mL at 37°C for 1 h. DNA was extracted using a commercial kit to obtain bacterial DNA (NucleoSpin^®^ TriPrep, Macherey-Nagel), according to the recommendations of the manufacturer. Intracanal exudate DNA was extracted by the boiling modified method (Queipo-Ortuño et al., 2008); the Eppendorf tubes containing the paper points and the RTF medium were boiled in a water bath for 10 minutes, followed by cooling down to -20°C for 10 minutes and were finally centrifuged for five minutes at 1000 rpm. PBMC bacterial DNA was extracted in a lysis buffer using lysozyme followed by the TE buffer and DNEasy isolation kit (QIAGEN Inc., Valencia, CA, USA), according to the manufacturer. The concentration and quality of DNA were confirmed by the 260:280 ratio in a spectrophotometer (Bio-Tek, Winooski, VT).

### qPCR Assay

Quantitative PCR (SteponePlus^®^; Applied Biosystems, Singapore) was carried out to determine bacterial loads and frequencies of detection of total bacteria, *P. gingivalis* and *P. endodontalis*. Primer sets are shown in **Table 1**. Briefly, total bacteria were quantified using specific primers targeting 16S ribosomal RNA (rRNA) gene previously published (Nadkarni et al., 2002); as well as *P. gingivalis*, which was identified by using previously validated 16S rRNA gene primers (Byrne et al., 2009). The primer set for targeting *P. gingivalis* included 16S rRNA from base 729 to 1192 (GenBank: L16492.1). For *P. endodontalis* identification, specific primers targeting the heat shock protein 60 were used (Hsp60 gene) (Sakamoto and Ohkuma, 2010). The Hsp60 gene sequence was obtained from GeneBank NIH genetic sequence database (GenBank: AB547580.1). Primer pairs were designed and assessed manually in the Primer Blast tool, considering the real-time PCR guide (BIO RAD). Which included having a melting temperature (Tm) between 50°C and 65°C, a GC content of 50–60%, to be limited to obtain an amplicon ranging from 75 to 200 bp. The selected primer pairs, targeting from base 84 to 201, were checked for specificity and cross-reactivity using Primer Blast and UniProt databases. Experimental validation included: firstly, the analysis of the molecular weight of the DNA fragments of three positive samples for *P. endodontalis* in agarose gel electrophoresis. Secondly, positive samples in step 1 were confirmed by Sanger sequencing (Macrogen, Seoul, Korea), showing 100% alignment with *P. endodontalis* (Hsp60 gene). Also, to determine the primer specificity, a dissociation curve analysis was performed, using temperatures between 60°C and 95°C for the recognition of non-specific product formation or contamination.

**Table 1:** Primers used to determine the bacterial loads and frequency of target bacteria.

Target bacteria	Target gene	Forward primer (5’-3’)	Reverse primer (5’-3’)
Total Bacteria	16S rRNA	TCCTACGGGAGGCAGCAGT	GGACTACCAGGGTATCTAATCCTGTT
*P. endodontalis*	Hsp 60	TATTGACAAGGCTGTGGCTACC	TTCTTCGTCCCCATTAGCCGA
*P. gingivalis*	16S rRNA	AGGCAGCTTGCCATACTGCG	ACTGTTAGTAACTACCGATGT

Each qPCR was performed using KAPA SYBR ^®^ Fast qPCR Kits (KAPA Biosystems, Woburn, MA, USA). Positive controls consisting of *P. gingivalis* ATCC 33277 and *P. endodontalis* ATCC 35406 DNA, and negative control (no DNA) were included in each experiment. Cycling conditions consisted of the following steps: 95°C for 3 min, 40 cycles: total bacteria 95°C for 15 sec and 60°C for 1 min; *P. gingivalis* 95°C for 3 sec and 58°C for 1 min; *P. endodontalis* 95°C for 30 seconds and 58°C for 1 min. As it was stated above, both *in silico* and experimental approaches were used for primers validation.

Absolute quantifications of total bacterial and individual species loads were carried out by comparing the threshold cycle (Dorn et al., 2002) values of the test samples to standard curves of known DNA copy numbers of *P. gingivalis* and *P. endodontalis* (**Supplementary Figure 1**). The lower and higher detection limits were 10^2^ and the 10^8^ DNA copy numbers, respectively. All experiments were performed with a linear quantitative detection range, **Supplementary Figure 1**. Bacterial loads were expressed as DNA copies/mg in the case of ALEOs, and DNA copies/μL in the case of intracanal exudates and PBMCs.

### Statistical Analysis

The sample size was calculated during a pilot study based on the positive detection of *P. endodontalis* in AAP versus SAP in ALEOs. Considering an 80% statistical power and 5% alpha error, a minimum of 23 samples in each group (SAP and AAP) were required.

Data distribution was evaluated by the Shapiro-Wilk test. Fisher’s exact Chi-square tests were used to compare the frequency of bacterial detection between two groups; Mann-Whitney test was used to compare bacterial loads between two groups; and McNemar’s and Spearman’s correlation tests were conducted to compare the associations in the frequencies of detection and bacterial loads, respectively between intracanal exudates and PBMCs within AP individuals. Analyses were performed using STATA 12^®^ (StataCorp LP, TX, USA). The level of statistical significance was p<0.05.

## Results

**Figure 1:**
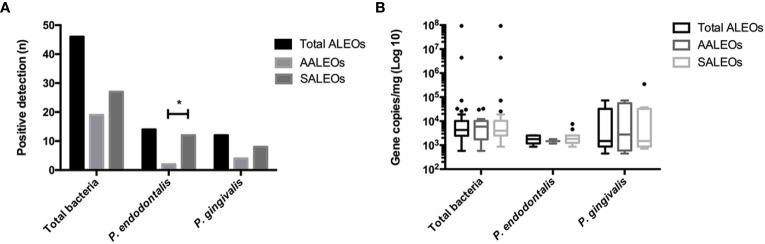
**(A)** Frequency of detection and **(B)** copy numbers of 16S rRNA gene of total bacteria and *P. gingivalis*, and Hsp60 gene of *P. endodontalis* in total ALEOs (n = 64) including AALEOs (n = 29) and SALEOs (n = 35). ALEOs Apical lesions of endodontic origin; AALEO asymptomatic apical lesions of endodontic origin; SALEOs symptomatic apical lesions of endodontic origin. *p < 0.05.

This study included a total of 126 individuals. Demographics and smoking habits are shown in **Table 2**. **Figures 1A, B** shows extraradicular infection rates for total bacteria, *P. endodontalis*, and *P. gingivalis*. The detection frequency, expressed as n (%), and bacterial loads expressed as medians (interquartile range) in ALEOs were 46 (70.8%) and 4521.6 (7789.1) DNA copy number/mg, respectively for total bacteria; 14 (21.5%) and 1789.7(1338.8) DNA copy number/mg for *P. endodontalis*; 12 (18.4%) and 1493.9 (26919.9) DNA copy number/mg for *P. gingivalis*. A higher frequency of detection was identified for *P. endodontalis* in symptomatic ALEOs compared to asymptomatic ALEOs (p = 0.003), while no significant differences were found in the detection frequencies of total bacteria and *P. gingivalis* or bacterial loads (p > 0.05). Healthy periodontal ligaments confirmed no bacterial detection.

**Table 2:** Demographic data and smoking habits of study groups.

Parameters	Tissue Samples		Intracanal (n = 39)	PBMCs	
	ALEOs (n = 64)	HPL (n = 9)		AP (n = 14)	Control (n = 14)
Age [years, median (IQR)]	38 (22.5)	22 (5)	25 (5)	24 (9.25)	22 (6.5)
Females (n, %)	27 (42.2%)	6 (66.7%)	18 (46.2%)	5 (35.7%)	4 (28.6%)
Smokers (n, %)	28 (44.4%)	2 (22%)	14 (35%)	9 (64.3%)	1 (7.1%)
Educational level (median)	Full high school	Full high school	Full high school	Full high school	Full high school

ALEOs, Apical lesions of endodontic origin; HPL, healthy periodontal ligament; PBMCs, Peripheral blood mononuclear cells; IQR, interquartile range.

**Figure 2:**
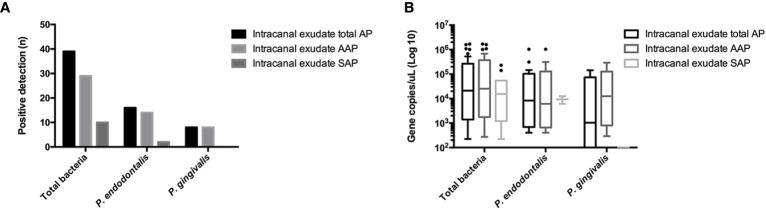
**(A)** Frequency of detection and **(B)** copy numbers of 16S rRNA gene of total bacteria and *P. gingivalis*, and Hsp60 gene of *P. endodontalis* in the root canal system exudates in AP (n = 39) including AAP (n = 29) and SAP (n = 10). AP Apical periodontitis; AAP asymptomatic apical periodontitis; SAP symptomatic apical periodontitis.

The frequency of detection and loads of total bacteria and *Porphyromonas* spp. in intracanal exudates are shown in **Figures 2A, B**. The frequencies of detection expressed as n (%) and bacterial loads expressed as median (interquartile range) DNA copy number/μL, respectively in AP were n=39 (100%) and 21089.2 (263450.7) for total bacteria; n=16 (41%) and 8263.9 (85001.8) for *P. endodontalis;* and n=8 (20.5%), 12538.9 (72957.3) for *P. gingivalis*. The frequency of detection and bacterial loads for *P. endodontalis* were 48.3% and 6127.9 (121214.7) in AAP, and 20%, and 9331.2 (6437.1) in SAP exudates, (p > 0.05). *P. gingivalis* was only identified in intracanal exudates from AAP with positive detection of n=8 (27.6%) and a bacterial load of 12538.9 (107919.3) DNA copy number/μL.

**Figure 3:**
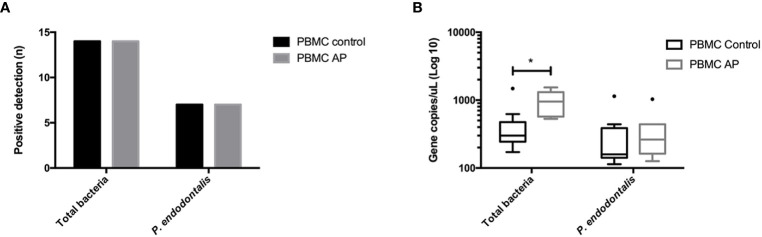
**(A)** Frequency of detection and **(B)** copy numbers of 16S rRNA gene of total bacteria and *P. gingivalis*, and Hsp60 gene of *P. endodontalis* in PMBCs from patients with AP (n = 14) and healthy subjects (n = 14). PBMCs Peripheral blood mononuclear cells; AP Apical periodontitis. *p < 0.05.

The frequency of bacterial DNA detection and loads in PBMCs are presented in **Figures 3A, B**. Bacteria were detected in 100% of the PBMC samples from AP and control groups. The total bacterial load was significantly higher in AP individuals compared to healthy controls (953.6 vs 300.6 median DNA copy number/μL respectively; p = 0.0002). On the other hand, *P. endodontalis* was detected in 50% of the samples of both, AP individuals and controls, showing an even higher frequency of detection than in intracanal exudates and apical lesions, though its bacterial loads were low, and no statistically significant differences were found between AP and healthy groups (p > 0.05). *P. gingivalis* was not detected in any of the evaluated PBMC samples (data not shown).

To explore the potential endodontic origin of the bacteria detected in PBMCs, the frequencies and loads of total bacteria, *P. endodontalis*, and *P. gingivalis* were associated between the intracanal samples and PBMCs within each AP individual (**Table 3**). Detection of total bacteria was positive in all intracanal exudates and PBMCs samples (14/14). *P. endodontalis* was identified in only two intracanal exudate samples (2/14) and seven PBMC samples (7/14) in AP individuals. From them, only one patient was positive for *P. endodontalis* at both, the intracanal exudate and the respective PBMCs. On the other hand, *P. gingivalis* was detected in three intracanal samples, though it was not detected in PBMCs. Regarding bacterial loads, no correlation was found between intracanal exudates and PBMCs (Spearman’s rho = 0.28, p = 0.33; data now shown).

**Table 3:** Endodontic/oral bacterial detection in intracanal exudates and respective PBMCs samples in AP individuals.

	PBMC (n = 14)					
Intracanal exudates (n = 14)	Total Bacteria		*P. endodontalis*		*P. gingivalis*	
Detection	Positive	Negative	Positive	Negative	Positive	Negative
**Positive**	14	0	1	1	0	3
**Negative**	0	0	6	6	0	11

PBMC, Peripheral blood mononuclear cells; AP, apical periodontitis; P. Porphyromonas. Detection expressed like positive and negative detection in intracanal exudates and PBMCs. p > 0.05.

## Discussion

ALEO is the result of a polymicrobial infection of the root canal system that causes local inflammation and destruction of the periapical tissues. Though bacteria have been largely assumed to be confined within the necrotic tooth canal, emerging evidence suggests that they can disseminate beyond the tooth structures (Liljestrand et al., 2016). Based on bacterial DNA detection, in the current study, we show a high frequency of extraradicular infection. Specifically, *P. endodontalis* was detected with a higher frequency in symptomatic compared to asymptomatic ALEO. Moreover, endodontic bacteria were detected in circulating mononuclear cells, whereas total bacterial loads were higher in PBMCs from AP compared to healthy individuals.

The presence of bacteria in the root canal system of teeth with AP has long been documented (Ricucci and Siqueira, 2010), but the concept of extraradicular infection is rather recent with only a few studies available. Given their high sensitivity, molecular techniques are widely being used to characterize the microbial communities (Rôças and Siqueira, 2008; Zhang et al., 2010; Rôças et al., 2011). Herein, we seek for unspecific oral bacteria and specific *Porphyromonas* spp. in ALEOs by qPCR. Identification of viable *Porphyromonas* spp. was formerly demonstrated through bacterial culture techniques in periapical abscesses (Van Winkelhoff et al., 1985). As part of the endodontic microbiome, Gram-negative species, such as *P. endodontalis* and *P. gingivalis*, are likely to have a clinical significance due to their wide repertoire of virulence factors (Sundqvist et al., 1989). Our results revealed high detection frequencies of total bacteria (70.8%), *P. gingivalis* (18.4%), and *P. endodontalis* (21.5%) in ALEOs. Specific identification of *P. endodontalis* and *P. gingivalis* with frequencies of 45% and 27% respectively, was also reported in ALEOs from persistent apical periodontitis based on qPCR (Zhang et al., 2010). The current study also explored the association between extraradicular infection and clinical symptoms, reporting a significantly higher detection frequency of *P. endodontalis* in ALEOs from SAP compared to AAP, while the detection frequencies and bacterial loads of *P. gingivalis* and total bacteria did not show differences. Though experimental mono-infection by *P. endodontalis* generates low pathogenicity compared to *P. gingivalis*, anaerobic mixed communities containing both species can trigger severe infective responses (Van Winkelhoff et al., 1985). Presumably, *P. endodontalis* might contribute to the bacterial mixed community to become more virulent, recruiting an enhanced immune response and giving rise to clinical symptoms. Therefore, it seems plausible that extraradicular translocation of *P. endodontalis* plays a relevant role in active forms of ALEOs.

The infection of the root canal system has been extensively characterized in AP (Gharbia et al., 1994; Fouad et al., 2002; Siqueira, 2002; Ricucci and Siqueira, 2010). Bacteria have been detected up to 100% within root canals from primary endodontic infection by molecular techniques (Conrads et al., 1997; Rôças and Siqueira, 2010). In line with this, intracanal samples of AP were all positive for bacterial DNA in our study. In earlier studies, the intracanal detection of *Porphyromonas* spp. revealed low recovery rates (9-23.9%) by culture methods (Hashioka et al., 1992; Baumgartner et al., 1999; Jacinto et al., 2003), though recent data based on molecular techniques report higher prevalence in primary endodontic infections (between 23.3-50% and 33-44%, respectively) (Tomazinho and Avila-Campos, 2007; Cao et al., 2012). Similarly, our results show detection frequencies of 41.0% for *P. endodontalis* and 20.5% for *P. gingivalis* in intracanal samples, confirming the relevant etiologic role of these black-pigmented anaerobic rods in AP (Dougherty et al., 1998; Baumgartner et al., 1999; de Oliveira et al., 2000).

Though the etiologic role of the intracanal bacterial infection is a matter of fact, its association with clinical symptoms remains unclear. Our results show that the detection frequencies and loads of total bacteria and *Porphyromonas* spp. in endodontic canals remained similar among AAP and SAP. Previous studies have reported comparable detection rates in root canals, especially for *P. endodontalis*, varying between 56% to 62% in AAP; and 40% to 69% in SAP (Van Winkelhoff et al., 1985; Rôças et al., 2002; Rôças and Siqueira, 2008; Rôças et al., 2011; Cardoso et al., 2016); while the available evidence of the detection of *P. gingivalis* ranges between 8-38% in SAP and 4-29% in AAP (Wang et al., 2010; Buonavoglia et al., 2013). The frequent intracanal detection of *Porphyromonas* spp. in association with AAP might be related to the confinement of the infection to the root canal. Importantly, in this condition bacteria gain no access to blood vessels as pulpal tissue is necrotic (Rôças and Siqueira, 2010). Moreover, our results showed that the relative detection of both, *P. endodontalis* and *P. gingivalis*, was higher in ALEOs than intracanal exudates from SAP; and conversely, they were higher in intracanal exudates than ALEOs in AAP; even though a direct comparison cannot be performed because ALEOs and intracanal exudates were obtained from different individuals based on their treatment indications, altogether these antecedents suggest that the transit to an acute periapical process and the consecutive onset of clinical symptoms might rely on the host’s inability to confine the endodontic pathogens -such as *P. endodontalis-* within the root canals and extraradicular infection might represent the first step for bacterial dissemination.

Given the anatomic relation of ALEOs with the bloodstream, it is plausible that inflammatory mediators, bacteria, and/or their products translocate from their endodontic sites to the systemic circulation (Garrido et al., 2019). Oral bacteria can be transported to distant tissues as free circulating DNA and/or internalized in immune cells (Martinez-Martinez et al., 2009; Ibrahiem et al., 2011), though the former is less probable because circulating oral bacteria are cleared within few minutes (Minasyan, 2019). Accordingly, we screened for bacterial DNA within PBMCs from AP and healthy individuals and found significantly higher total bacterial loads in the former, suggesting that ALEOs create a favorable environment for the overgrowth of microorganisms and their systemic dissemination. Moreover, *P. endodontalis* DNA was also detected in PBMCs from individuals in both groups (50%). In fact, a previous study reported the carriage of bacterial DNA (16S rDNA) in PBMCs from arthritis and control individuals (Ibrahiem et al., 2011). Despite no studies are available in AP, a pathophysiological role for blood dendritic cells in systemic dissemination of periodontal pathobionts to atherosclerotic plaques was proposed, particularly of *P. gingivalis* (Carrion et al., 2012). Herein we failed to detect *P. gingivalis* in PBMCs, probably because our study individuals were free from periodontitis. Instead, *P. endodontalis* might analogously translocate to distant sites from oral/endodontic sites *via* PBMCs and has also the ability to invade endothelial cells *in vitro* (Dorn et al., 2002). Additionally, bacterial carriage has been associated with mononuclear cell activation, production of pro-inflammatory cytokines, prolonged survival of circulating monocytes, and further risk of future complications of systemic non-communicable diseases, including cirrhosis and hemodialysis (Hazzah et al., 2015; Rodriguez-Laiz et al., 2019). These results are in line with the higher inflammatory burden of serum markers reported in patients with ALEOs (Garrido et al., 2019). Altogether, these preliminary studies support that oral/endodontic microorganisms can translocate from their habitats to systemic circulation and reach distant organs using mononuclear cells as Trojan horses. The capacity for intracellular survival of bacteria within phagocytes is likely a critical factor facilitating the dissemination. Some bacteria can hide within autophagosomes, where they protected themselves from phagocytic killing (Berthelot and Wendling, 2020).

Despite the abovementioned bacterial translocation in AP, no association was found between the presence and loads of bacteria between endodontic canals and PBMCs within the study individuals. Oral bacteria and specifically *P. endodontalis* might coexist in various oral habitat, including subgingival sites and periodontal pockets, as well as oral mucosa (*i.e.* tonsils and tongue), where they can also be phagocytosed by mononuclear cells and reach the general circulation (Lombardo Bedran et al., 2012; Scapoli et al., 2015).

Although bacterial cultures are considered the reference method to determine the presence of microorganisms (Zweitzig et al., 2013), molecular analysis is more sensitive, especially in anaerobic conditions, such as necrotized root canals (Baumgartner, 2004). Particularly, *Porphyromonas* is considered fastidious genera and culture methods may underestimate their frequency (Gomes and Herrera, 2018). Also, no viable oral bacteria can be recovered from peripheral lesions in most studies (Martinez-Martinez et al., 2009; Chhibber-Goel et al., 2016), whereas PCR has proven to be highly sensitive to detect bacterial translocation (Kane et al., 1998). Instead, bacterial DNA is much more likely to disseminate from oral niches through blood and can trigger and perpetuate inflammation after bacterial clearance.

Summarizing, total bacteria and specific *Porphyromonas* spp. were frequently found in endodontic canals and extra-radicular infection in AP patients. Extraradicular bacteria and specifically, detection of *P. endodontalis*, associated with symptomatic forms of the disease and might represent a first step in the dissemination process. Also, *P. endodontalis* was frequently detected in PBMCs from study individuals and higher total bacterial loads were found in AP, supporting a role for these cells in the bacterial carriage during AP.

## Data Availability Statement

The raw data supporting the conclusions of this article will be made available by the authors, without undue reservation.

## Ethics Statement

The studies involving human participants were reviewed and approved by Ethics-Scientific Committee of the Central Metropolitan Health Service (N 2017/70) and from the Faculty of Dentistry, Universidad de Chile (N 2016/08). Written informed consent to participate in this study was provided by the participants’ legal guardian/next of kin.

## Author Contributions

MH, MG, AH: Conceptualization and design. MG, MB: Data curation. MH: Funding acquisition. MH, AF, MB, AH: Formal analysis. MB, JA, AH: Laboratory experiments. MH: Project administration. AH: Design the figures. MH: Resources. MH: Software. MG, MH: Supervision. MB, AF, MH: Writing manuscript. All authors contributed to the article and approved the submitted version.

## Funding

This study was funded by FONDECYT 1160741 and 1200098. AF is a recipient of scholarship CONICYT 21181377, from Chilean Government.

## Conflict of Interest

The authors declare that the research was conducted in the absence of any commercial or financial relationships that could be construed as a potential conflict of interest.

## Supplementary Material

Supplementary Figure 1 Quantitative PCR standard curves obtained for total bacteria **(A)**, P. gingivalis **(B)** and P. endodontalis **(C)** quantification. The regression line from the 10-fold dilutions curve was used to determine the copy number of all samples. All reactions were performed in duplicate.

## References

[B1] Baumgartner J. C. Watkins B. J. Bae K.-S. Xia T. (1999). Association of black-pigmented bacteria with endodontic infections. J. Endodontics 25, 413–415. 10.1016/S0099-2399(99)80268-4 10530240

[B2] Baumgartner J. C. (2004). Microbiological and molecular analysis of endodontic infections. Endodontic Topics 7, 35–51. 10.1111/j.1601-1546.2004.00061.x

[B3] Berthelot J. M. Wendling D. (2020). Translocation of dead or alive bacteria from mucosa to joints and epiphyseal bone-marrow: facts and hypotheses. Joint Bone Spine 87, 31–36. 10.1016/j.jbspin.2019.01.004 30677505

[B4] Buonavoglia A. Latronico F. Pirani C. Greco M. F. Corrente M. Prati C. (2013). Symptomatic and asymptomatic apical periodontitis associated with red complex bacteria: clinical and microbiological evaluation. Odontology 101, 84–88. 10.1007/s10266-011-0053-y 22143381

[B5] Byrne S. J. Dashper S. G. Darby I. B. Adams G. G. Hoffmann B. Reynolds E. C. (2009). Progression of chronic periodontitis can be predicted by the levels of Porphyromonas gingivalis and Treponema denticola in subgingival plaque. Oral. Microbiol. Immunol. 24, 469–477. 10.1111/j.1399-302X.2009.00544.x 19832799

[B6] Cao H. Qi Z. Jiang H. Zhao J. Liu Z. Tang Z. (2012). Detection of Porphyromonas endodontalis, Porphyromonas gingivalis and Prevotella intermedia in primary endodontic infections in a Chinese population. Int. Endodontic J. 45, 773–781. 10.1111/j.1365-2591.2012.02035.x 22429191

[B7] Cardoso F. G. D. R. Chung A. Martinho F. C. Camargo C. H. R. Carvalho C. A. T. Gomes B. P. F. D. A.. (2016). Investigation of Bacterial Contents From Persistent Endodontic Infection and Evaluation of Their Inflammatory Potential. Braz. Dental J. 27, 412–418. 10.1590/0103-6440201600520 27652703

[B8] Carrion J. Scisci E. Miles B. Sabino G. J. Zeituni A. E. Gu Y.. (2012). Microbial carriage state of peripheral blood dendritic cells (DCs) in chronic periodontitis influences DC differentiation, atherogenic potential. J. Immunol. (Baltimore Md. 1950) 189, 3178–3187. 10.4049/jimmunol.1201053 PMC3459682 22891282

[B9] Chhibber-Goel J. Singhal V. Bhowmik D. Vivek R. Parakh N. Bhargava B.. (2016). Linkages between oral commensal bacteria and atherosclerotic plaques in coronary artery disease patients. NPJ Biofilms Microbiomes 2, 7. 10.1038/s41522-016-0009-7 28649401 PMC5460270

[B10] Conrads G. Gharbia S. E. Gulabivala K. Lampert F. Shah H. N. (1997). The use of a 16S rDNA directed PCR for the detection of endodontopathogenic bacteria. J. Endodontics 23, 433–438. 10.1016/S0099-2399(97)80297-X 9587296

[B11] de Oliveira J. C. M. Siqueira J. F. Jr Alves G. B. Hirata R. Jr Andrade A. F. (2000). Detection of Porphyromonas endodontalis in infected root canals by 16S rRNA gene-directed polymerase chain reaction. J. Endodontics 26, 729–732. 10.1097/00004770-200012000-00016 11471643

[B12] Dorn B. Harris L. Wujick C. Vertucci F. Progulske-Fox A. (2002). Invasion of vascular cells in vitro by Porphyromonas endodontalis. Int. Endodontic J. 35, 366–371. 10.1046/j.0143-2885.2001.00489.x 12059938

[B13] Dougherty W. Bae K. Watkins B. Baumgartner J. (1998). Black-pigmented bacteria in coronal and apical segments of infected root canals. J. Endodontics 24, 356–358. 10.1016/S0099-2399(98)80134-9 9641113

[B14] Fouad A. F. Barry J. Caimano M. Clawson M. Zhu Q. Carver R.. (2002). PCR-based identification of bacteria associated with endodontic infections. J. Clin. Microbiol. 40, 3223–3231. 10.1128/JCM.40.9.3223-3231.2002 12202557 PMC130810

[B15] Garrido M. Cardenas A. M. Astorga J. Quinlan F. Valdes M. Chaparro A.. (2019). Elevated Systemic Inflammatory Burden and Cardiovascular Risk in Young Adults with Endodontic Apical Lesions. J. Endodontics 45, 111–115. 10.1016/j.joen.2018.11.014 30711165

[B16] Gharbia S. E. Haapasalo M. Shah H. N. Kotiranta A. Lounatmaa K. Pearce M. A.. (1994). Characterization of Prevotella intermedia and Prevotella nigrescens isolates from periodontic and endodontic infections. J. Periodontol. 65, 56–61. 10.1902/jop.1994.65.1.56 7907659

[B17] Gomes B. Herrera D. (2018). Etiologic role of root canal infection in apical periodontitis and its relationship with clinical symptomatology. Braz. Oral. Res. 32, 82–110. 10.1590/1807-3107bor-2018.vol32.0069 30365610

[B18] Gutmann J. L. Baumgartner J. C. Gluskin A. H. Hartwell G. R. Walton R. E. (2009). Identify and define all diagnostic terms for periapical/periradicular health and disease states. J. Endodontics 35, 1658–1674. 10.1016/j.joen.2009.09.028 19932340

[B19] Hashioka K. Yamasaki M. Nakane A. Horiba N. Nakamura H. (1992). The relationship between clinical symptoms and anaerobic bacteria from infected root canals. J. Endodontics 18, 558–561. 10.1016/S0099-2399(06)81214-8 1298793

[B20] Hazzah W. A. Hashish M. H. El-Koraie A. F. Ashour M. S. Abbass A. A. (2015). Circulating bacterial DNA fragments in chronic hemodialysis patients. Saudi J. Kidney Dis. Transpl. 26, 1300–1304. 10.4103/1319-2442.168689 26586077

[B21] Hsiao W. W. Li K. L. Liu Z. Jones C. Fraser-Liggett C. M. Fouad A. F. (2012). Microbial transformation from normal oral microbiota to acute endodontic infections. BMC Genomics 13, 345. 10.1186/1471-2164-13-345 22839737 PMC3431219

[B22] Ibrahiem B. E. El Haliem N. F. El-Khouly N. El-Bazz W. F. Al-Raoof M. A. Said Z. N. (2011). Detection of bacterial DNA in peripheral blood mononuclear cells of patients with reactive arthritis. Egyptian J. Immunol. 2, 67–76. 23082472

[B23] Jacinto R. Gomes B. Ferraz C. Zaia A. Filho F. S. (2003). Microbiological analysis of infected root canals from symptomatic and asymptomatic teeth with periapical periodontitis and the antimicrobial susceptibility of some isolated anaerobic bacteria. Oral. Microbiol. Immunol. 18, 285–292. 10.1034/j.1399-302X.2003.00078.x 12930519

[B24] Kane T. D. Alexander J. W. Johannigman J. A. (1998). The detection of microbial DNA in the blood: a sensitive method for diagnosing bacteremia and/or bacterial translocation in surgical patients. Ann. Surg. 227, 1–9. 10.1097/00000658-199801000-00001 9445103 PMC1191165

[B25] Liljestrand J. M. Mantyla P. Paju S. Buhlin K. Kopra K. A. Persson G. R.. (2016). Association of Endodontic Lesions with Coronary Artery Disease. J. Dental Res. 95, 1358–1365. 10.1177/0022034516660509 27466397

[B26] Lombardo Bedran T. B. Marcantonio R. A. C. Spin Neto R. Alves Mayer M. P. Grenier D. Spolidorio L. C.. (2012). Porphyromonas endodontalis in chronic periodontitis: a clinical and microbiological cross-sectional study. J. Oral. Microbiol. 4, 10123. 10.3402/jom.v4i0.10123 PMC3253302 22232719

[B27] Martinez-Martinez R. E. Abud-Mendoza C. Patino-Marin N. Rizo-Rodriguez J. C. Little J. W. Loyola-Rodriguez J. P. (2009). Detection of periodontal bacterial DNA in serum and synovial fluid in refractory rheumatoid arthritis patients. J. Clin. Periodontol. 36, 1004–1010. 10.1111/j.1600-051X.2009.01496.x 19929953

[B28] Minasyan H. (2019). Sepsis: mechanisms of bacterial injury to the patient. Scandinavian J. Trauma Resuscitation Emergency Med. 27, 19–19. 10.1186/s13049-019-0596-4 PMC6376788 30764843

[B29] Nadkarni M. A. Martin F. E. Jacques N. A. Hunter N. (2002). Determination of bacterial load by real-time PCR using a broad-range (universal) probe and primers set. Microbiology 148, 257–266. 10.1099/00221287-148-1-257 11782518

[B30] Queipo-Ortuño M. I. De Dios Colmenero J. Macias M. Bravo M. J. Morata P. (2008). Preparation of bacterial DNA template by boiling and effect of immunoglobulin G as an inhibitor in real-time PCR for serum samples from patients with brucellosis. Clin. Vaccine Immunol. 15, 293–296. 10.1128/CVI.00270-07 18077622 PMC2238042

[B31] Reichert S. Haffner M. Keysser G. Schafer C. Stein J. M. Schaller H. G.. (2013). Detection of oral bacterial DNA in synovial fluid. J. Clin. Periodontol. 40, 591–598. 10.1111/jcpe.12102 23534379

[B32] Ricucci Siqueira (2010). Biofilms and apical periodontitis: study of prevalence and association with clinical and histopathologic findings. J. Endodontics 36, 1277–1288. 10.1016/j.joen.2010.04.007 20647081

[B33] Rôças Siqueira (2008). Root Canal Microbiota of Teeth with Chronic Apical Periodontitis. J. Clin. Microbiol. 46, 3599. 10.1128/JCM.00431-08 18768651 PMC2576597

[B34] Rôças Siqueira (2010). Identification of bacteria enduring endodontic treatment procedures by a combined Reverse Transcriptase–Polymerase Chain reaction and Reverse-Capture Checkerboard approach. J. Endodontics 36, 45–52. 10.1016/j.joen.2009.10.022 20003934

[B35] Rocas I. N. Siqueira J. F. Jr. (2011). In vivo antimicrobial effects of endodontic treatment procedures as assessed by molecular microbiologic techniques. J. Endod. 37, 304–310. 10.1016/j.joen.2010.11.003 21329812

[B36] Rôças Siqueira (2018). Frequency and levels of candidate endodontic pathogens in acute apical abscesses as compared to asymptomatic apical periodontitis. PloS One 13, e0190469. 29293651 10.1371/journal.pone.0190469 PMC5749828

[B37] Rôças Siqueira Andrade Uzeda D. (2002). Identification of selected putative oral pathogens in primary root canal infections associated with symptoms. Anaerobe 8, 200–208. 10.1006/anae.2002.0431

[B38] Rôças Siqueira Debelian (2011). Analysis of Symptomatic and Asymptomatic Primary Root Canal Infections in Adult Norwegian Patients. J. Endodontics 37, 1206–1212. 10.1016/j.joen.2011.05.026 21846535

[B39] Rodriguez-Laiz G. P. Zapater P. Melgar P. Alcazar C. Franco M. Gimenez P.. (2019). Bacterial DNA translocation contributes to systemic inflammation and to minor changes in the clinical outcome of liver transplantation. Sci. Rep. 9, 835. 10.1038/s41598-018-36904-0 30696924 PMC6351615

[B40] Sahingur S. E. Xia X. J. Alamgir S. Honma K. Sharma A. Schenkein H. A. (2010). DNA from Porphyromonas gingivalis and Tannerella forsythia induce cytokine production in human monocytic cell lines. Mol. Oral. Microbiol. 25, 123–135. 10.1111/j.2041-1014.2009.00551.x 20331800 PMC4017325

[B41] Sakamoto M. Ohkuma M. (2010). Usefulness of the hsp60 gene for the identification and classification of Gram-negative anaerobic rods. J. Med. Microbiol. 59, 1293–1302. 10.1099/jmm.0.020420-0 20671088

[B42] Sakko M. Tjaderhane L. Rautemaa-Richardson R. (2016). Microbiology of Root Canal Infections. Primary Dental J. 5, 84–89. 10.1308/205016816819304231 28826437

[B43] Scapoli L. Girardi A. Palmieri A. Martinelli M. Cura F. Lauritano D.. (2015). Quantitative analysis of periodontal pathogens in periodontitis and gingivitis. J. Biol. Regulators Homeostatic Agents 29, 101–110. 26511188

[B44] Siqueira (2002). Endodontic infections: Concepts, paradigms, and perspectives. Oral. Surg. Oral. Med. Oral. Pathol. Oral. Radiol. Endodontol. 94, 281–293. 10.1067/moe.2002.126163 12324780

[B45] Sunde P. T. Tronstad L. Eribe E. R. Lind P. O. Olsen I. (2000). Assessment of periradicular microbiota by DNA-DNA hybridization. Endodontics Dental Traumatol. 16, 191–196. 10.1034/j.1600-9657.2000.016005191.x PMC7194203 11202881

[B46] Sundqvist G. Johansson E. Sjögren U. (1989). Prevalence of black-pigmented bacteroides species in root canal infections. J. Endodontics 15, 13–19. 10.1016/S0099-2399(89)80092-5 2607261

[B47] Tomazinho L. F. Avila-Campos M. J. (2007). Detection of Porphyromonas gingivalis, Porphyromonas endodontalis, Prevotella intermedia, and Prevotella nigrescens in chronic endodontic infection. Oral. Surg. Oral. Med. Oral. Pathol. Oral. Radiol. Endodontol. 103, 285–288. 10.1016/j.tripleo.2006.05.010 17234549

[B48] Van Winkelhoff A. Carlee A. De Graaff J. (1985). Bacteroides endodontalis and other black-pigmented Bacteroides species in odontogenic abscesses. Infection Immun. 49, 494–497. 10.1128/IAI.49.3.494-497.1985 PMC261188 4030089

[B49] Veloso P. Fernandez A. Terraza-Aguirre C. Alvarez C. Vernal R. Escobar A.. (2020). Macrophages skew towards M1 profile through reduced CD163 expression in symptomatic apical periodontitis. Clin. Oral. Investig. 24, 4571–4581. 10.1007/s00784-020-03324-2 32444919

[B50] Wang Q. Zhou X.-D. Zheng Q.-H. Wang Y. Tang L. Huang D.-M. (2010). Distribution of Porphyromonas gingivalis fimA Genotypes in Chronic Apical Periodontitis Associated with Symptoms. J. Endodontics 36, 1790–1795. 10.1016/j.joen.2010.08.018 20951289

[B51] Zhang S. Wang Q. Q. Zhang C. F. Soo I. (2010). Identification of dominant pathogens in periapical lesions associated with persistent apical periodontitis. Chin. J. Dental Res. 13, 115–121. 21264361

[B52] Zweitzig D. R. Riccardello N. M. Morrison J. Rubino J. Axelband J. Jeanmonod R.. (2013). Measurement of microbial DNA polymerase activity enables detection and growth monitoring of microbes from clinical blood cultures. PloS One 8, e78488. 10.1371/journal.pone.0078488 24155986 PMC3796490

