# Supplementary figures and images for: Systemic and Extraradicular Bacterial Translocation in Apical Periodontitis
Source: Front Cell Infect Microbiol. 2021 Mar 19;11:649925. doi: 10.3389/fcimb.2021.649925 (PMC8017189; doi:10.3389/fcimb.2021.649925)

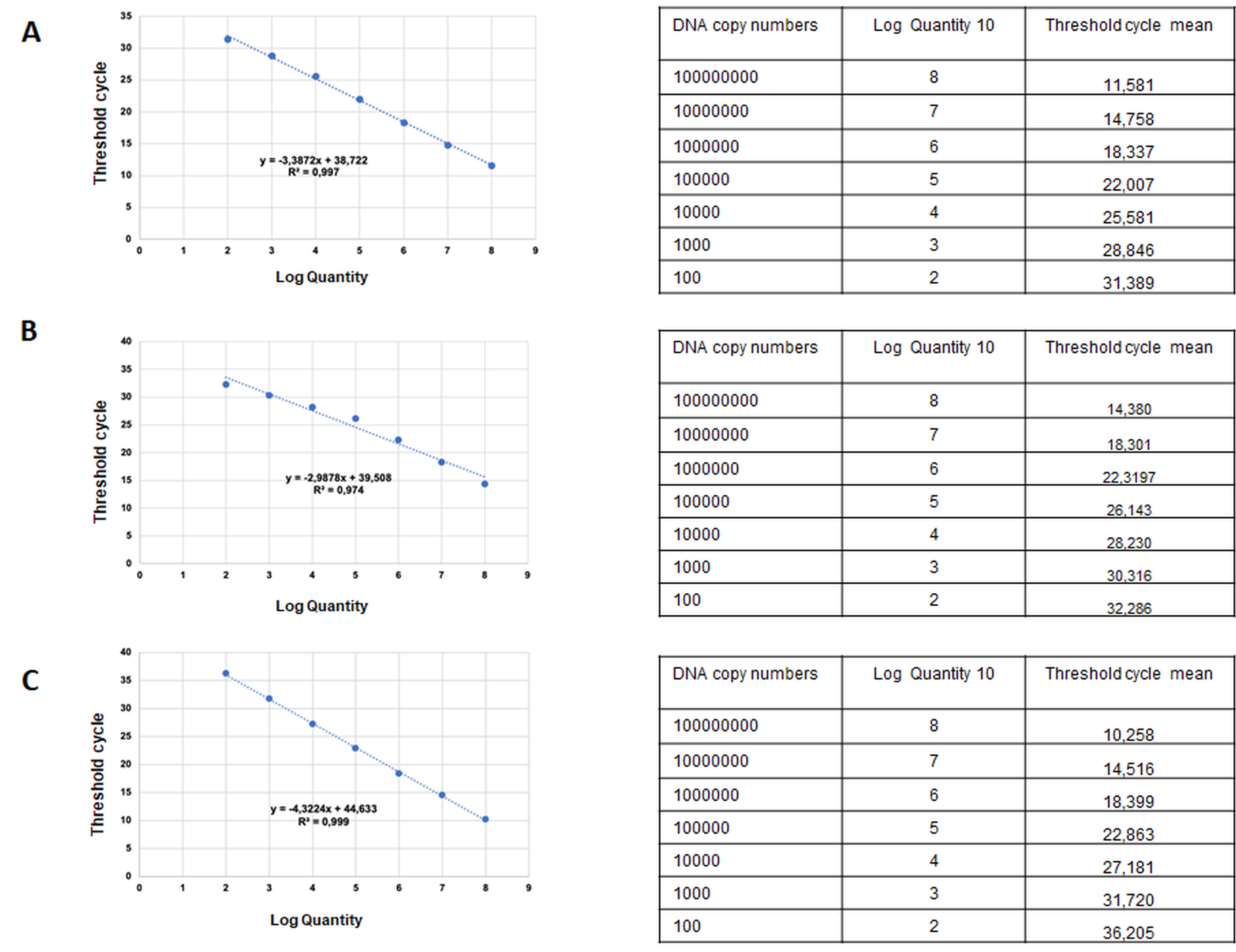

Supplement: Supplementary Figure 1 — Quantitative PCR standard curves obtained for total bacteria (A), P. gingivalis (B) and P. endodontalis (C) quantification. The regression line from the 10-fold dilutions curve was used to determine the copy number of all samples. All reactions were performed in duplicate. [file Image_1.tif]
